# Systematic Review and Meta-Analysis of the Efficacy of Interleukin-1 Receptor Antagonist in Animal Models of Stroke: an Update

**DOI:** 10.1007/s12975-016-0489-z

**Published:** 2016-08-15

**Authors:** Sarah K. McCann, Fala Cramond, Malcolm R. Macleod, Emily S. Sena

**Affiliations:** Centre for Clinical Brain Sciences, University of Edinburgh, Chancellor’s Building, 49 Little France Crescent, Edinburgh, EH16 4SB UK

**Keywords:** Systematic review, Meta-analysis, Interleukin-1 receptor antagonist, Neuroprotection, Focal cerebral ischaemia, Experimental validity

## Abstract

Interleukin-1 receptor antagonist (IL-1 RA) is an anti-inflammatory protein used clinically to treat rheumatoid arthritis and is considered a promising candidate therapy for stroke. Here, we sought to update the existing systematic review and meta-analysis of IL-1 RA in models of ischaemic stroke, published in 2009, to assess efficacy, the range of circumstances in which efficacy has been tested and whether the data appear to be confounded due to reported study quality and publication bias. We included 25 sources of data, 11 of which were additional to the original review. Overall, IL-1 RA reduced infarct volume by 36.2 % (95 % confidence interval 31.6–40.7, *n* = 76 comparisons from 1283 animals). Assessments for publication bias suggest 30 theoretically missing studies which reduce efficacy to 21.9 % (17.3–26.4). Efficacy was higher where IL-1 RA was administered directly into the ventricles rather than peripherally, and studies not reporting allocation concealment during the induction of ischaemia reported larger treatment effects. The preclinical data supporting IL-1 RA as a candidate therapy for ischaemic stroke have improved. The reporting of measures to reduce the risk of bias has improved substantially in this update, and studies now include the use of animals with relevant co-morbidities.

**Electronic supplementary material:**

The online version of this article (doi:10.1007/s12975-016-0489-z) contains supplementary material, which is available to authorized users.

## Introduction

In recent years, systematic review and meta-analysis have been used to provide less biased summaries of the published evidence supporting the efficacy of candidate drugs for stroke. The initial drivers for this effort were to help select drugs to be tested in clinical trials [1] and to identify important gaps in the evidence. For example, a systematic review of the efficacy of hypothermia in animal stroke studies [2] demonstrated high headline efficacy, across a range of circumstances, but also illustrated that the impact of pethidine (commonly used to manage shivering in humans undergoing awake hypothermia) on efficacy was not known. These findings led firstly to targeted animal experiments exploring the impact of pethidine [3] and then informed the design of the EuroHYP-1 clinical trial of hypothermia for acute ischaemic stroke [4].

An unintended consequence of this approach has been to establish the prevalence and impact of risks of bias in the animal literature modelling stroke. Initial work suggested a worryingly low prevalence of measures which might reduce the risk of bias such as randomisation and blinding and that studies which did not report such measures gave inflated estimates of treatment effects [5]. This informed the development of good practice guidelines for stroke research [6] and preclinical research more generally [7, 8]. Subsequent investigation showed that, far from being an extreme example of a highly biased research field, in vivo stroke researchers perform at least as well as researchers in other neuroscience domains and better than research published from leading UK institutions [7, 9]. Further, a review of reporting quality for publications in the journal *Stroke* describing in vivo research reveals an apparent improvement in reporting since the *Stroke* good laboratory practice (GLP) guidelines were published [10]. Whether this was caused by the adoption of GLP guidelines, changes in editorial policy or other factors is not known.

In 2006, we conducted a systematic review and meta-analysis of the effects of interleukin-1 receptor antagonist (IL-1 RA) in animal models of ischaemic stroke [11]. This suggested substantial efficacy but also identified a number of potential shortcomings in the supporting animal literature: there was significant heterogeneity between studies, the range of conditions under which efficacy was tested was narrow, study quality was modest when scored against established checklists and there was evidence consistent with a substantial publication bias. Specifically, there was a lack of evidence at times of administration beyond 180 min, of testing in animals with co-morbidities including hypertension or diabetes and of testing in larger animals.

That publication led to a letter [12] to the journal editor raising concerns about the utility of an aggregate quality “score” and about the importance attached in our review to the demonstration of efficacy in animals with co-morbidities. Subsequently, we have focussed in our systematic reviews on the prevalence of individual risk of bias items rather than calculating an overall score, but a lower efficacy in animals with co-morbidities has been demonstrated for a number of candidate neuroprotective drugs [5, 13].

IL-1 RA remains a promising drug for the treatment of stroke. Subsequent to our initial publication, there have been reports that it may modify the immune response following severe traumatic brain injury [14] and subarachnoid haemorrhage [15]. Clinical evaluation of IL-1 RA for the treatment of both ischaemic and haemorrhagic stroke is ongoing: three phase-II randomised controlled trials have been completed, one is ongoing and another is planned to start in 2018 [16]. The main findings in two of the completed studies suggest it is well tolerated in stroke patients and there are no safety concerns [15, 16]. To our knowledge, no phase-III trials in ischaemic stroke are currently under development.

Against this background, we set out to update our existing systematic review and meta-analysis of the efficacy of IL-1 RA in experimental stroke. As well as providing a summary of current data for efficacy, we were also interested to see whether there had been an increase in the range of circumstances under which efficacy has been tested and reported and whether there was an increase in the quality of reporting of studies published since our initial review.

## Methods

### Search Strategy

We searched PubMed, Embase, BIOSIS and Web of Science Core Collection for [(interleukin 1 receptor antagonist) OR (IL-1 RA) OR (IL1RA) OR (IL1-RA) OR (Anakinra)] AND [(stroke) OR (ischemia) OR (cerebrovascular) OR (middle cerebral artery) OR (MCA) OR (ACA) OR (anterior cerebral artery) OR (MCAO)] AND [(Hooijmans et al. PubMed animal filter [17]) OR (de Vries et al. Embase animal filter update [18])] NOT [(coronary) OR (myocardial)]. We restricted the date of publication to post-2005, and the search was completed in February 2016. Results were screened independently by title and abstract in the SyRF screening application (http://app.syrf.org.uk/) by up to three reviewers (minimum 0.66 agreement required for inclusion; FC, ESS and SKM). Full texts of included articles were then screened by two reviewers (ESS and SKM) with discrepancies resolved through discussion.

### Inclusion Criteria and Outcome Measures

We included data describing the effects of IL-1 RA compared to a control group receiving vehicle or no treatment in whole live animal models of focal cerebral ischaemia. We included any mode and route of delivery of IL-1 RA (e.g. transgenic, viral vector, peripheral) at any time point and frequency. The primary endpoint was infarct area or volume, and secondary endpoints were neurobehavioural scores and mortality.

### Data Extraction

Two reviewers independently extracted study design, quality and outcome data for each included comparison (ESS and SKM). We abstracted from studies the time of first drug administration, cumulative drug dose in the first 24 h of administration (recorded in mg/kg for peripheral and total weight [μg] for central administration), route of drug delivery, type (permanent/temporary/thrombotic) and method of ischaemic occlusion, time to outcome measurement, anaesthetic used, whether or not animals were ventilated during surgery, method of infarct measurement, publication status, and the species, strain and sex of animals used. Where a control group served more than one treatment group, the size of the control group used for meta-analysis was adjusted accordingly. Where outcomes from the same group of animals were reported at different time points, the last time point was extracted. Where data were presented graphically, digital measuring software was used, and where this was not possible, authors were contacted seeking the original data. Where outcome data extracted digitally by the two independent reviewers differed by <10 %, an average of the two values was taken. Data differing by >10 % and any other discrepancies were resolved through discussion with a third reviewer (MRM).

### Range of Evidence

We assessed the range of evidence against the updated Stroke Therapy Academic Industry Roundtable (STAIR) criteria [19]: (1) evidence from two or more laboratories, (2) from two or more species, (3) from animals with co-morbidities, (4) from male and female animals, (5) from both permanent and temporary models of ischaemia, (6) testing at least two doses of the drug, (7) with some doses given at least 1 h after vessel occlusion, (8) testing using a feasible route of drug delivery, (9) use of both histologic and behavioural outcomes, (10) outcome measured at least 4 weeks after vessel occlusion, (11) from species other than rodents, (12) interaction studies with medications commonly used in stroke patients and (13) use of relevant biomarker endpoints.

### Quality of Evidence

We assessed the susceptibility to bias of each publication using the CAMARADES study quality checklist [20] adapted to include relevant items from the updated STAIR criteria [19]: (1) peer reviewed publication, (2) control of temperature, (3) randomisation of group allocation, (4) blinded induction of ischaemia, (5) blinded assessment of outcome, (6) avoidance of anaesthetics with marked intrinsic neuroprotective properties, (7) use of animals with co-morbidities (e.g. hypertension, diabetes), (8) sample size calculation, (9) statement of compliance with animal welfare requirements, (10) statement of potential conflicts of interest, (11) physiological monitoring during stroke induction (in addition to control of temperature, e.g. blood pressure, gases), (12) prespecified inclusion and exclusion criteria, (13) reporting of animals excluded from analysis, (14) reporting of study funding, and (15) injury confirmed via laser Doppler or perfusion imaging.

The range and quality of evidence items from the updated STAIR recommendations were also extracted from the publications included in our original review.

### Analysis

Our analysis plan was prespecified in a study protocol, published online at (https://drive.google.com/file/d/0B5x-sP1A05kgWWYtX09RejBockE/view). For infarct and neurobehavioural outcomes, we calculated a normalised mean difference for each comparison, and for mortality, the odds ratio [21]. For each outcome, comparisons were combined using random-effects modelling with a restricted maximum likelihood (REML) estimate of between-study variance. Where different measures of neurobehavioral outcome were reported from the same cohort of animals at the same time point, we combined these (pre-nested) comparisons using fixed-effect meta-analysis (nesting) and used this summary estimate in the random-effects model.

We used meta-regression to investigate possible sources of heterogeneity including components of the study quality checklist and study design characteristics, and a significance level of *p* < 0.05 was set for each test.

We tested for the presence and extent of publication bias using funnel plots, Egger’s test and trim and fill [22, 23].

Because of concerns that meta-regression may be underpowered in detecting important differences between studies due to aspects of study design, we also analysed these differences using partitioning of heterogeneity as a sensitivity analysis. Sensitivity analyses were performed using DerSimonian and Laird random-effects meta-analysis, and stratified meta-analysis was used to investigate sources of heterogeneity. Stratifications were considered in two domains: study design and study quality, with each domain tested at *p* < 0.05 overall. A Holm-Bonferroni adjusted critical *p* value was calculated to account for the number of parameters tested within each domain; *p* < 0.003 for study design and *p* < 0.007 for study quality.

Heterogeneity is described using *Q* (heterogeneity statistic), tau^2^ (estimation of between-study variance), residual *I*^2^ (the percentage of the residual variation that is attributable to between-study heterogeneity) and adjusted *R*^2^ (adj *R*^2^; the proportion of between-study variance explained by the covariate).

Statistical analyses were performed using Stata Statistical Software: Release 13 (StataCorp LP, College Station, TX) or Microsoft Access 2013.

## Results

Four hundred and thirty-three publications were identified electronically, of which 10 met our inclusion criteria. Requests to authors for unpublished data provided one further manuscript [24]. In total, 11 studies were added to our original dataset. We identified one publication [25] describing studies included in our original review as a conference abstract and unpublished data (Clark 2005 and Clark 2006 in Banwell et al. [11], confirmed through personal communication); these original data are therefore excluded from the current analysis. We identified one publication from the original review where median data were reported (an exclusion criterion in the current review), and this study is also excluded from the current analysis [26]. Our updated dataset includes 25 studies in total (Supp Fig. 1); study characteristics are shown in Table 1.

**Table 1:** Study characteristics

Study	Species	No. of animals	Dose	Time of first admin (min)	Anaesthetic	Type of ischaemic model	Route of delivery	Modality	Outcome measure
Betz et al. 1995 [31]	Rat	12	9.1 ng/g	−7200	Isoflurane	Permanent	ICV	Vector	Infarct volume
Boutin et al. 2001 [32]	Mouse	23	5 μg	−30	Halothane	Temporary	ICV	Protein	Infarct volume
Clark et al. 2008 [25]	Rat	14	–	0	Halothane	Temporary	IV	Protein	Infarct volume
Clausen et al. 2016 [33]	Mouse	140	10^7^ cells	−60,480–30	Isoflurane	Permanent	IV	Tg, Tg BM cells	Infarct volume, neurobehaviour
Craft et al. 2006 [34]	Mouse	17	6 μg	8640	Halothane	Temporary	ICV	Protein	Infarct volume, neurobehaviour
Denes et al. 2014 [35]	Rat, mouse	52	100 mg/kg	90–120	Isoflurane	Temporary	IP, SubCut	Protein	Infarct volume, neurobehaviour
Garcia et al. 1995 [36]	Rat	37	400 mg/kg	0	Ketamine	Permanent	IV	Protein	Infarct volume, neurobehaviour, mortality
Girard et al. 2014 [37]	Rat	20	100 mg/kg	0–1440	Isoflurane	Temporary	SubCut	Protein	Infarct volume, neurobehaviour
Greenhalgh et al. 2010 [38]	Rat	17	100 mg/kg	0	Isoflurane	Temporary	SubCut	Protein	Infarct volume
Le Feuvre et al. 2003 [39]	Mouse	14	10 μg	0	Halothane	Temporary	ICV	Protein	Infarct volume
Loddick et al. 1996 [40]	Rat	99	10–20 mg/kg	−30–30	Halothane	Permanent	ICV	Protein	Infarct volume
Mao et al. 2000 [41]	Mouse	16	–	–7200	Isoflurane	Temporary	ICV	Vector	Infarct volume
Maysami et al. 2015 [27]	Mouse	220	200 mg/kg	30	Isoflurane, halothane, tribromoethanol	Permanent, temporary, thrombotic	SubCut	Protein	Infarct volume, neurobehaviour, mortality
McColl et al. 2007 [42]	Mouse	25	300 mg/kg	−30	Halothane	Temporary	IP	Protein	Infarct volume, neurobehaviour
Mulcahy et al. 2003 [43]	Rat	84	20 μg	0–180	Halothane	Temporary	ICV	Protein	Infarct volume
Pradillo et al. 2012 [44]	Rat	38	25–50 mg/kg	90–270	Isoflurane	Temporary	SubCut	Protein	Infarct volume
Pradillo et al. 2016 [24]	Rat	54	25–100 mg/kg	270	Isoflurane	Temporary	SubCut	Protein	Infarct volume, neurobehaviour
Relton et al. 1992 [45]	Rat	24	20 μg	−30	Halothane	Permanent	ICV	Protein	Infarct volume
Relton et al. 1996 [46]	Rat	210	50–1000 mg/kg	0–60	Halothane	Permanent	IV, SubCut	Protein	Infarct volume
Stroemer et al. 1997 [47]	Rat	72	5–7.5 μg	0	Halothane	Permanent	Stereotactic	Protein	Infarct volume
Touzani et al. 2002 [48]	Mouse	16	5 μg	−30	Halothane	Temporary	ICV	Protein	Infarct volume
Tsai et al. 2003 [49]	Rat	20	0.77 ng/g	–	Chloral hydrate	Temporary	ICV	Vector	Infarct volume
Xia et al. 2014 [50]	Rat	60	5–20 mg/kg	180–720	Unknown	Temporary	IV	Protein	Infarct volume, neurobehaviour
Yang et al. 1997 [51]	Mouse	12	2 ng/g	−7200	Isoflurane	Temporary	ICV	Vector	Infarct volume
Yang et al. 1999 [52]	Mouse	28	–	−7200	Isoflurane	Temporary	ICV	Vector	Infarct volume

Abbreviations: *admin* administration (in relation to the onset of ischaemia), *ICV* intracerebroventricular, *IV* intravenous, *IP* intraperitoneal, *SubCut* subcutaneously, *Tg* transgenic, *BM *bone marrow

The range of evidence met 11 of a possible 13 STAIR criteria assessed. Criteria newly met in the current study are evidence from animals with co-morbidities which include aged, aged corpulent (a model of metabolic syndrome) and acute infection with pneumonia or LPS, and outcome assessed at 4 weeks post-ischaemia. The dataset now includes experiments where IL-1 RA is administered up to 6 days after induction of ischaemia, with 12 comparisons where administration is more than 3 h post-stroke. The number of neurobehavioural outcomes reported increased from 1 to 33 comparisons. All studies published post-2009 administered IL-1 RA peripherally including intravenous, intraperitoneal and subcutaneous routes. In our original review, over half of studies used central administration via intracerebroventricular or intracerebral stereotactic routes. Relevant biomarker endpoints including MRI assessment of injury have been reported. Although IL-1 RA has been studied in animals also treated with tissue plasminogen activator (tPA), no in vivo interaction studies with medications commonly used by stroke patients such as statins, blood pressure-lowering medication and aspirin were identified. Evidence is still lacking in female animals and in species other than rodents.

Overall, the number of study quality items met is greater in studies published post-2009 (median 11.5/15 interquartile range [IQR] 9.75–12) than pre-2009 (median 6/15 IQR 5–7; Table 2).

**Table 2:** Change over time: A comparison of the data prior to the publication of our 2009 review, afterwards and with all data pooled

		Pre-2009	Post-2009	Overall
No. publications		17	8	25
Animals/paper		42.5	75.1	53
Infarct volume	Effect size (95 % CI)	37.5 % (30.3–44.7)	36.15 % (31.8–40.7)	36.5 % (31.6–41.3)
	*I* ^2^ (%)	87	72	82
	# experiments	39	37	76
	# animals	709	574	1283
Neurobehavioural outcome	Effect size (95 % CI)	24.8 % (−8.7–58.3)	37.1 % (29.7–44.5)	35.8 % (28.2–43.5)
	*I* ^2^ (%)	67	58	58
	# experiments	4	29	33
	# animals	51	422	473
Mortality	Odds ratio (95 % CI)	0.5 (0.04–5.8)	1.1 (0.5–2.8)	1.03 (0.5–2.4)
	*I* ^2^	–	–	–
	# experiments	1	9	10
	# animals	26	201	227
Median quality (/15) (interquartile range)		6 (5–7)	11.5 (9.8–12)	7.0 (5–7)
Random allocation to group (%)		5.9	87.5	32.0
Blinded induction of ischaemia (%)		11.8	50.0	24.0
Blinded assessment of outcome (%)		29.4	87.5	48.0
Sample size calculation (%)		0.0	37.5	12.0
Statement of potential conflict of interest (%)		0.0	87.5	28.0
Prespecified exclusion of animals (%)		11.8	62.5	28.0
Explanation of exclusions (%)		11.8	62.5	28.0

In particular, the proportion of studies reporting randomisation, blinded induction of ischaemia, blinded assessment of outcome, prespecified exclusion criteria and animal exclusions increased substantially. Clear differences are also evident in the proportion of studies using co-morbid animal models and those reporting a sample size calculation (Supp Table 1). Pre-2009, no studies reported a statement regarding possible conflicts of interest. Post-2009, seven out of eight studies included a statement; of these seven, one reported no conflict while six made disclosures. Use of laser Doppler or perfusion imaging to confirm ischaemic injury was assessed as a quality item; however, alternative methods of confirmation were reported in some studies: through behavioural observation in one study and visually (microscopically) in two studies.

Infarct volume was reported in 76 comparisons from 1283 animals, neurobehavioural score in 98 (33 nested) comparisons from 473 animals and mortality in 10 comparisons from 227 animals. These data met our prespecified criterion for a minimum 30 % increase in the number of independent comparisons required to justify an updated meta-analysis (original dataset 44 infarct volume, 1 neurobehavioral and 2 mortality comparisons).

**Fig. 1:**
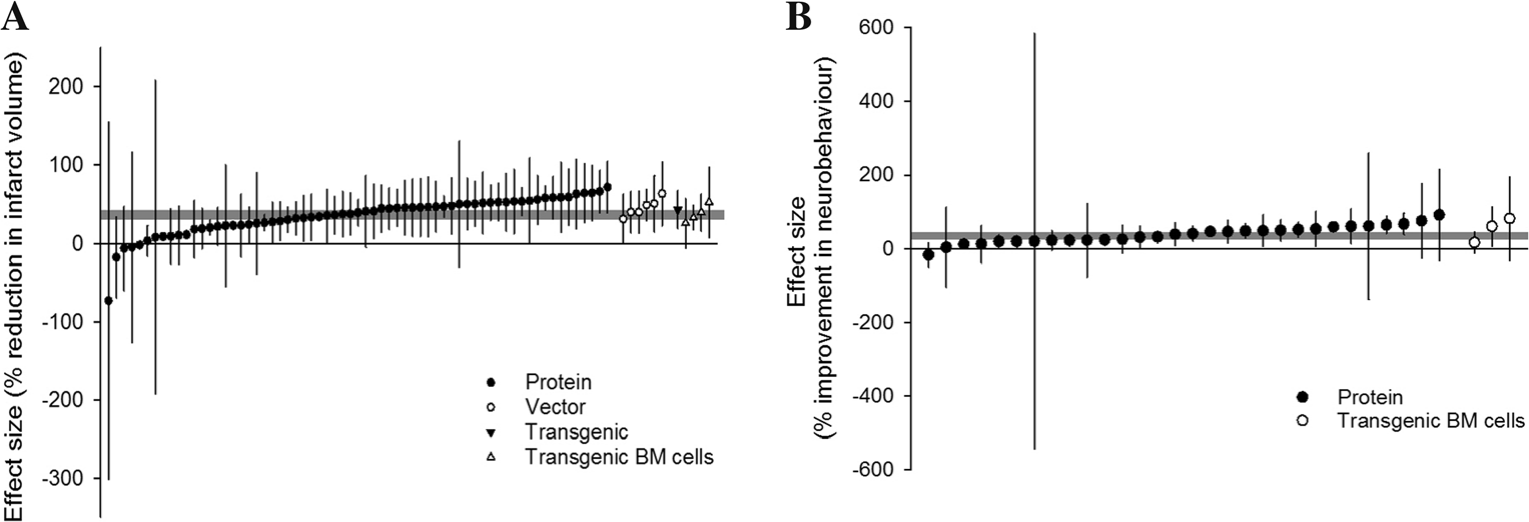
Effect of IL-1 RA on **a** infarct volume and **b** neurobehavioural outcomes. Individual nested comparisons grouped according to the mode of IL-1 RA delivery and ranked according to effect. *Shaded grey bars* represent 95 % CI of global estimate of efficacy. *Vertical error bars* represent 95 % CI for individual estimates

Overall, IL-1 RA reduced our primary outcome, infarct volume, by 36.2 % (95 % confidence interval [CI] 31.6–40.7). Administration of IL-1 RA in protein form resulted in a 35.5 % (30.3–40.7) reduction, administration of IL-1 RA transgenic bone marrow (BM) cells a 34.7 % (15.9–53.6) reduction and vector transfection a 44.7 % (29.9–59.6) reduction. One comparison involved transgenic mice overexpressing IL-1 RA resulting in a 43.2 % (19.1–67.3) reduction in infarct size (Fig. 1a).

**Fig. 2:**
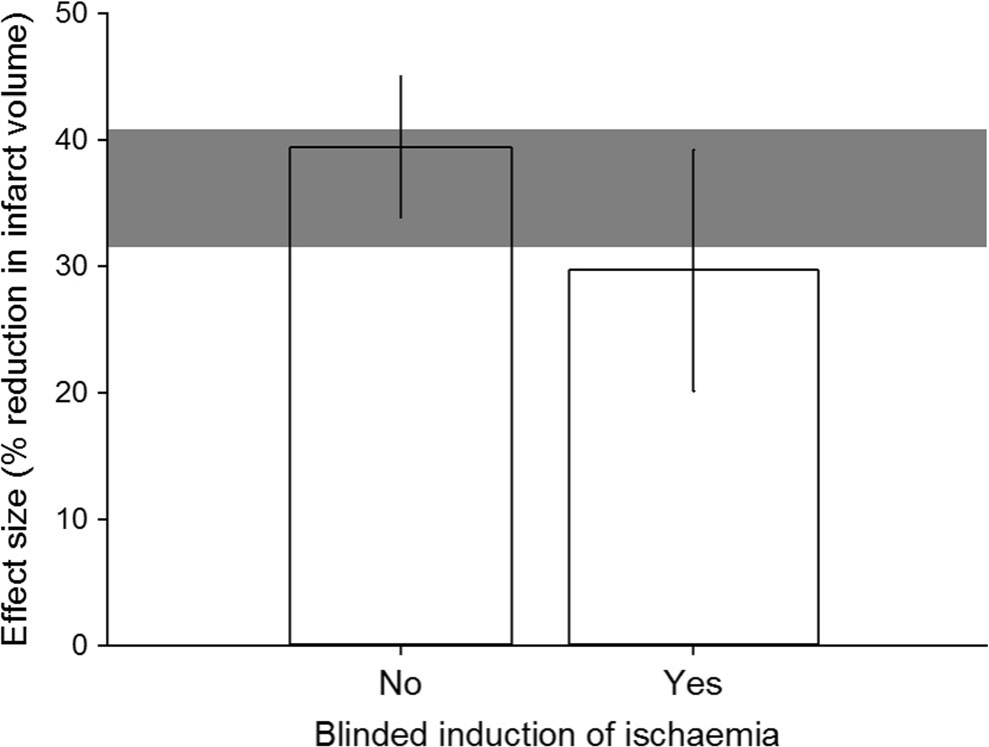
Correction of measured infarct volume for presence of blinded induction of ischaemia. *Shaded grey bar* represents 95 % CI of global estimate of efficacy. *Vertical error bars* represent 95 % CI for individual estimates. Width of each vertical bar reflects square root of number of animals contributing to that comparison

For study quality, we observed greater reduction in infarct volume in studies that *did not* report that investigators were blinded to treatment allocation during the induction of ischaemia (*p* = 0.045, tau^2^ = 181.6, *I*^2^ = 82.4 %, adj *R*^2^ = 3.2 %, Fig. 2). Other potential sources of bias that did not account for a significant proportion of heterogeneity were reporting of randomisation to group, blinded assessment of outcome, prespecified exclusion criteria, reasons for excluding animals, sample size calculation and statement of potential conflict of interest.

Due to uncertainty around the timing and effective dose achieved in transgenic and transfection studies, analyses of study design characteristics are restricted to 19 sources describing 65 experiments where IL-1 RA was administered in protein form. Mode of IL-1 RA delivery (peripheral or central delivery) is not a significant source of heterogeneity (*p* = 0.412), and therefore, data were analysed together.

**Fig. 3:**
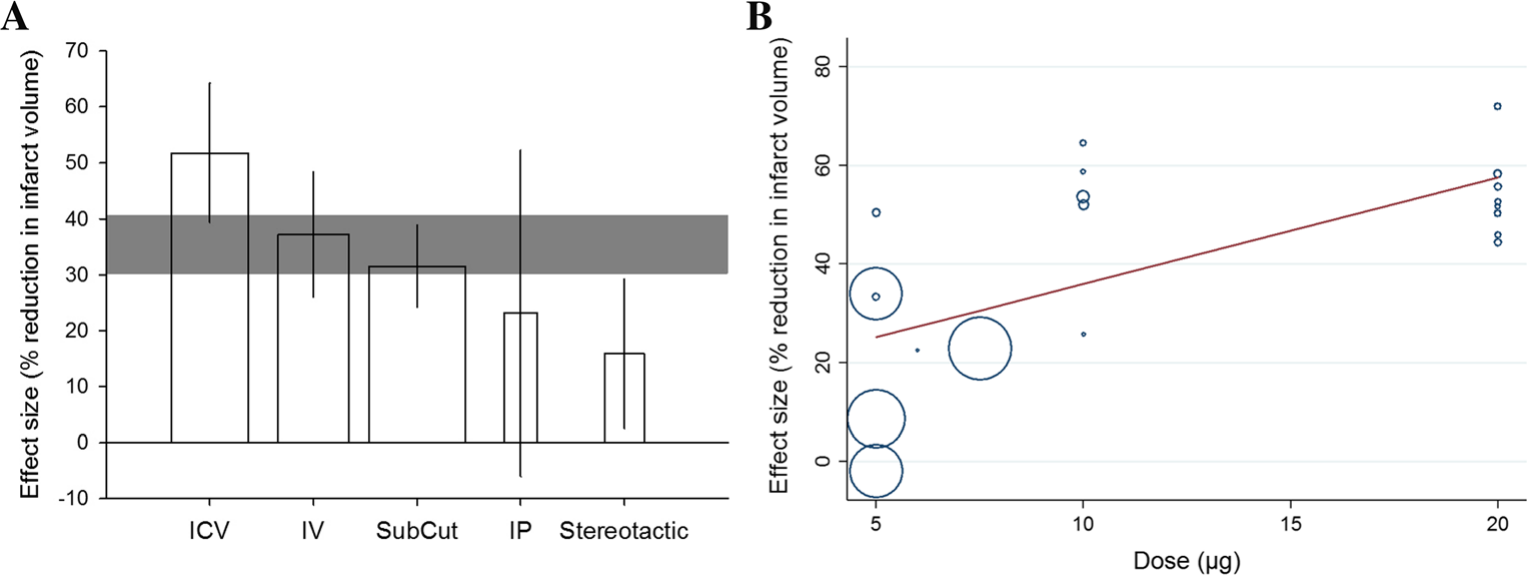
Heterogeneity in the effect of IL-1 RA on infarct volume is in part explained by **a** the route of delivery (*x-axis*). *Vertical error bars* represent 95 % CI for individual estimates. Width of each vertical bar reflects square root of number of animals contributing to that comparison (*ICV* intracerebroventricular, *IV* intravenous, *SubCut* subcutaneous, *IP* intraperitoneal). **b** Effect of IL-1 RA dose on estimate of efficacy. Size of points reflect the precision of each comparison (inverse of within-study variance). Stratification by dose accounts for significant proportion of heterogeneity observed between studies (*p* = 0.005)

We observed substantial heterogeneity in this dataset (tau^2^ = 231.9, *I*^2^ = 83.7 %) that was explained, in part, by two of the variables investigated with univariate meta-regression. Firstly, for route of delivery, studies using intracerebroventricular administration reported the greatest magnitude of effect (*p* = 0.0003, tau^2^ = 121.3, *I*^2^ = 77.54 %, adj *R*^2^ = 43.3 %). Large effects were also observed with the more clinically relevant peripheral routes of delivery, intravenous and subcutaneous (Fig. 3a). Secondly, dose-response relationships for central (intracerebroventricular, stereotactic) and peripheral (intravenous, intraperitoneal, subcutaneous) administration were analysed separately. Dose is a significant source of heterogeneity in experiments where IL-1 RA was administered centrally (*p* = 0.005, tau^2^ = 194.8, *I*^2^ = 89.6 %, adj *R*^2^ = 45.2 %) with larger reductions in infarct volume evident at higher doses (Fig. 3b).

Effect sizes for data stratified by publication date (pre- or post-2009) were similar with less statistical heterogeneity observed in more recent studies: we observed a reduction in infarct volume pre-2009 of 36.1 % (27.9–44.2), tau^2^ = 279.4 and *I*^2^ = 88.1 % versus 35.0 % (28.2–41.7), tau^2^ = 159.4 and *I*^2^ = 74.2 % post-2009, *p* = 0.97.

Variables that do not contribute significantly to heterogeneity include the following: species and sex of animals, time of IL-1 RA administration, whether single, multiple or continuous administration was used, whether infarct volume calculation involved a correction for oedema, method of infarct quantification, presence of co-treatments, co-morbidity studied, method of induction of ischaemia, type of ischaemia, anaesthetic used during model induction and whether mechanical ventilation was used, and time of outcome assessment relative to model induction.

**Fig. 4:**
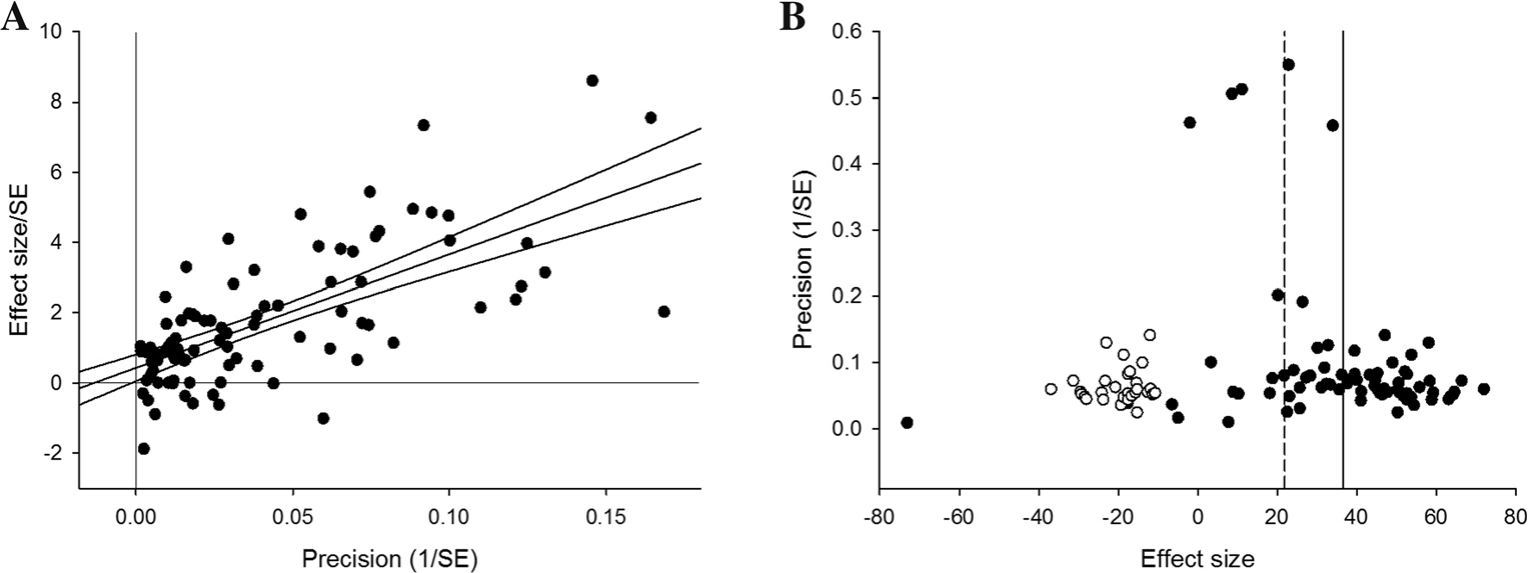
Publication bias in infarct volume estimates assessed by **a** Egger’s regression, showing regression line with 95 % CI, and **b** trim and fill analysis, showing the distribution of published study outcomes (*filled circles*) and imputed outcomes (*unfilled circles*). The *solid vertical line* represents the global estimate of efficacy and the *dashed line* the adjusted reduction in infarct volume when theoretically missing studies are incorporated

Funnel plot asymmetry is detected with Egger’s test (*p* < 0.001), suggesting the presence of publication bias (Fig. 4a). Trim and fill analysis imputed the presence of 30 “missing” experiments, with an adjusted reduction in infarct volume of 21.9 % (17.3–26.4, Fig. 4b), 14.3 % lower than before adjustment.

Overall, IL-1 RA improves neurobehavioural measures by 35.9 % (28.2–43.5; *n* = 33). No improvement was observed in experiments where IL-1 RA transgenic BM cells were administered (*n* = 3, *p* = 0.200) (Fig. 1b). Neurobehavioural measures were categorised as tests of motor/sensory behaviours, social interaction/anxiety/depressive behaviours or thermal nociception. Using this categorisation, the type of neurobehavioural test is not a significant source of heterogeneity (post hoc analysis; *p* = 0.4480). Most experiments (28/33) tested motor/sensory outcomes, and further analyses are restricted to these data due to the divergent biology underlying the behaviours tested in the remaining outcomes. Only experiments where IL-1 RA was administered in protein form were investigated for sources of heterogeneity (27 comparisons); in all of these experiments, IL-1 RA was administered peripherally.

**Fig. 5:**
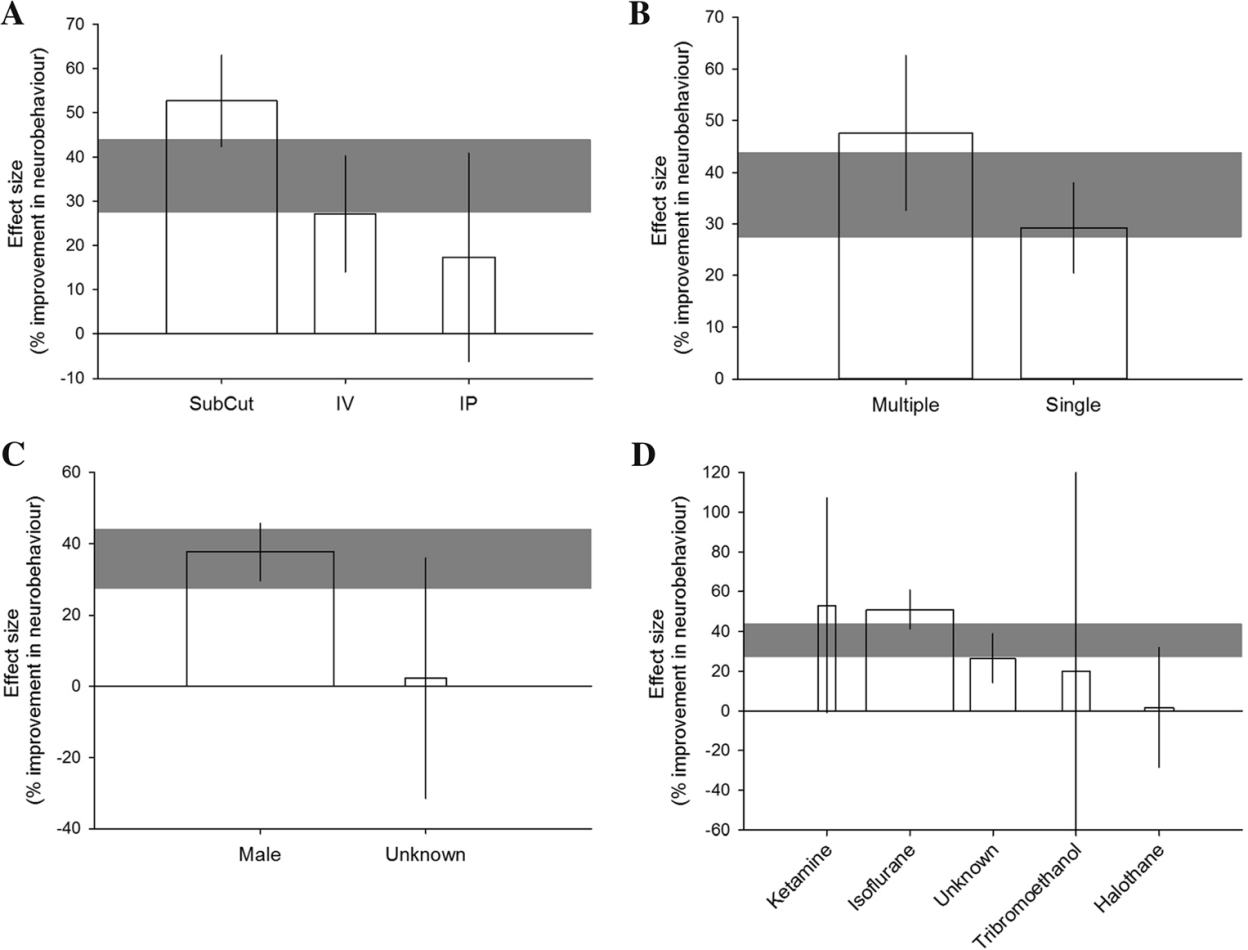
Heterogeneity in the effect of IL-1 RA on motor/sensory neurobehavioural outcomes is in part explained by **a** the route of delivery, **b** the number of doses administered, **c** the sex of the animals used and **d** the anaesthesia used during induction of ischaemia. *Shaded grey bars* represent 95 % CI of global estimate of efficacy. *Vertical error bars* represent 95 % CI for individual estimates. Width of each vertical bar reflects square root of number of animals contributing to that comparison

For motor/sensory behaviours, there is an improvement in outcome of 35.7 % (27.5–43.9; tau^2^ = 219.1, *I*^2^ = 63.8 %, *n* = 27). Route of delivery accounted significantly for this heterogeneity (*p* = 0.0008, tau^2^ = 71.8, *I*^2^ = 32.6 %, adj *R*^2^ = 67.2 %) with the greatest improvement seen with subcutaneous administration (Fig. 5a). Greater effects are also observed in experiments that administered multiple rather than single doses of IL-1 RA (*p* = 0.018, tau^2^ = 133.1, *I*^2^ = 49.9 %, adj *R*^2^ =39.2 %; Fig. 5b). Sex of the animals is a significant source of heterogeneity (*p* = 0.040, tau^2^ = 186.6, *I*^2^ = 61.3 %, adj *R*^2^ = 14.8 %), with no effect seen in experiments where the sex of the animal was not reported (Fig. 5c). The anaesthetic used during induction of ischaemia also contributes to heterogeneity (*p* = 0.0023, tau^2^ = 62.6, *I*^2^ = 31.7 %, adj *R*^2^ = 71.4 %). The greatest effect is seen in studies using isoflurane while there is no effect in those using ketamine, tribromoethanol or halothane (Fig. 5d). In addition to the effects of anaesthesia, post-operative analgesia can affect stroke outcome in rodents. Only two of the included studies reported using an analgesic (buprenorphine); therefore, we were unable to assess the impact of this variable on the recorded outcomes.

We further subdivided motor/sensory behavioural measures into the more specific categories: gross neurological score (*n* = 26), skilled movement task (*n* = 4) or sensorimotor asymmetry test (*n* = 7). Post hoc analysis revealed that type of motor/sensory measure was not a significant source of heterogeneity (*p* = 0.8182). Other variables not contributing significantly to heterogeneity are species of animals, dose and time of IL-1 RA administration, co-morbidity studied, method of induction of ischaemia, type of ischaemia and time of outcome assessment relative to model induction. Only one comparison involved a co-treatment (tPA), and for all comparisons, it was unknown whether mechanical ventilation was used; therefore, these variables were not analysed.

**Fig. 6:**
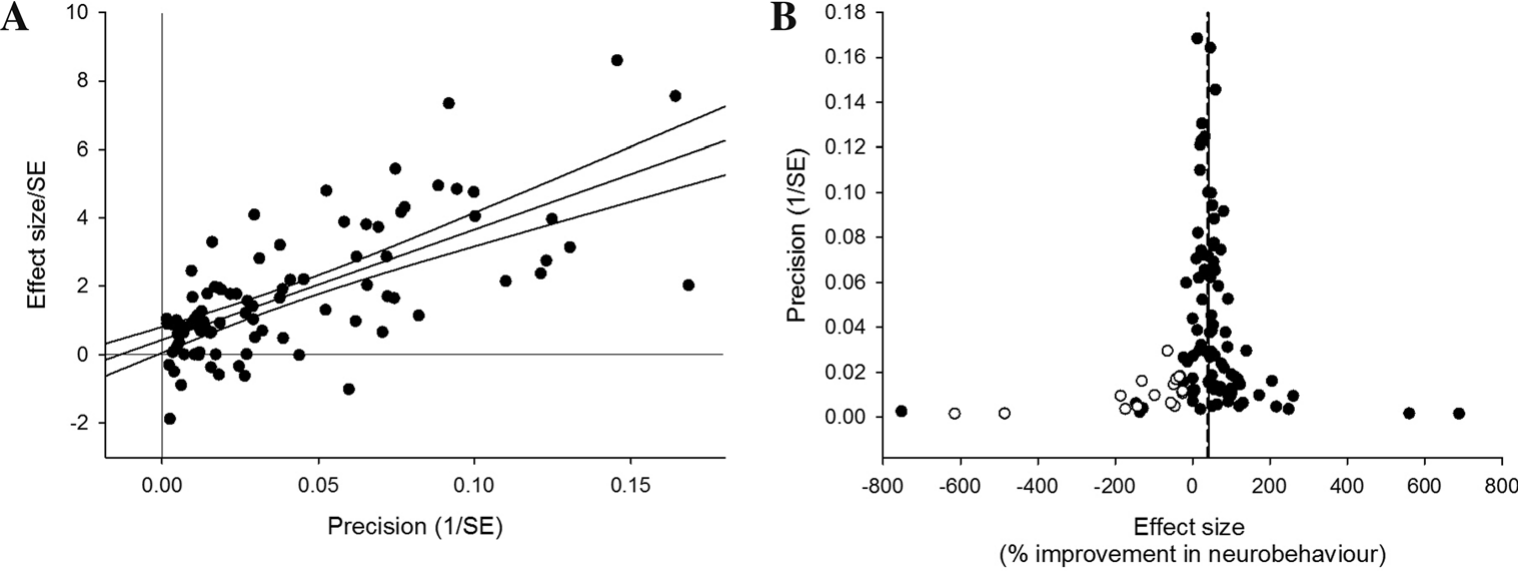
Publication bias in neurobehavioural outcome estimates assessed by **a** Egger’s regression, showing regression line with 95 % CI, and **b** trim and fill analysis, showing the distribution of published study outcomes (*filled circles*) and imputed outcomes (*unfilled circles*). The *solid vertical line* represents the global estimate of efficacy and the *dashed line* the adjusted reduction in infarct volume when theoretically missing studies are incorporated

Egger’s test suggests significant funnel plot asymmetry (*p* = 0.028) (Fig. 6a). Trim and fill analysis imputed the presence of 18 “missing” experiments, with improvement in neurobehavioural outcome adjusted from 41.4 % (34.9–47.9) down to 38.6 % (31.9–45.3) (Fig. 6b). These values differ from the estimate of efficacy calculated using meta-regression due to use of a moment-based rather than REML estimate of between-study variance.

Mortality was unaffected by administration of IL-1 RA with an odds ratio of 1.03 (0.45–2.38), *n* = 10, 227 animals, *Q* = 2.87 and *p* = 0.97. Mortality was not analysed further due to the limited data.

### Sensitivity Analyses

We performed stratified meta-analysis, rather than meta-regression, as a sensitivity analysis to assess the impact of study quality and design on infarct volume using an alternative statistical method. For study quality, studies reporting a formal calculation of study size reported larger treatment effects than those that did not (*Q* = 13.1, df = 1, *p* = 0.0003) (Supp Fig. 2A). For study design, we similarly observed larger effects with intracerebroventricular delivery of IL-1 RA (*Q* = 125.6, df = 4, *p* < 10^−25^) (Supp Fig. 2B) and a dose response for central drug administration (*Q* = 68.8, df = 2, *p* < 10^−14^) (Supp Fig. 2C). In addition, we observed other variables to account for significant sources of heterogeneity. We observed the largest effects in studies using ketamine anaesthesia (*Q* = 53.2, df = 4, *p* < 10^−10^) (Supp Fig. 2D), in thrombotic models of ischaemia (*Q* = 13.0, df = 2, *p* = 0.002) (Supp Fig. 2E) and where ischaemia was induced via thrombin injection (*Q* = 24.5, df = 3, *p* < 10^−4^) (Supp Fig. 2F) and in studies which reported correcting for oedema in infarct quantification (*Q* = 53.9, df = 2, *p* < 10^−11^) (Supp Fig. 2G). We observed an inverse dose response for peripheral drug delivery (*Q* = 60.8, df = 3, *p* < 10^−12^) (Supp Fig. 2H) and a significant but unclear relation between the time of drug administration and effect (*Q* = 72.3, df = 4, *p* < 10^−14^) (Supp Fig. 2I).

## Discussion

Treatment with IL-1 RA leads to substantial improvements in outcome in preclinical models of ischaemic stroke, whether measured as reduced infarct volume or improved neurobehavioural outcome. The range of evidence supporting the administration of IL-1 RA for treatment of focal ischaemic stroke has increased substantially since our previous systematic review and meta-analysis. Discussion with researchers in the field suggests that this has been due to deliberate efforts to test efficacy in circumstances identified as requiring further evidence. IL-1 RA has now been tested in animals with a range of co-morbidities, at times of administration beyond 180 min, with outcomes assessed up to 28 days after injury and where it is administered via a clinically plausible route. Current phase-II trials in the UK are investigating subcutaneous IL-1 RA as the intravenous formulation is no longer manufactured [16]. Our data suggests efficacy is maintained with subcutaneous delivery. The co-morbidities tested were corpulent rats and those with pneumonia or treated with LPS (as a surrogate for the response to infection). Importantly, aged animals have also been tested. In our primary analysis, these co-morbidities were not a significant source of heterogeneity suggesting that that the efficacy of IL-1 RA is maintained in spite of them; however, efficacy has yet to be tested in animals with hypertension and in animals other than rodents.

There were striking improvements in study quality since our 2009 review; the median number of quality checklist items scored increased from 6 of a possible 15 (IQR 5–7) to 11.5 (IQR 9.75–12), with substantial improvements across risk of bias items. This is consistent with the improvements observed in the reporting of in vivo research in the journal *Stroke* [10] and, importantly, was only associated with a small (and not significant) reduction in the observed efficacy. There were other interesting changes, including a substantial increase in the average number of animals reported in each paper, which increased from ∼42 to ∼75. This may reflect the increased use of power calculations.

When we planned this review, we changed the study quality and range of evidence items in response to the updated STAIR recommendations [19]. To address concerns over the utility of an aggregate quality “score”, we have instead identified seven items, identified as fundamentals of good scientific enquiry [8, 19], and analysed the impact of these individually. The only study quality measure that accounted for a significant proportion of the observed heterogeneity was allocation concealment during the induction of injury, where studies which did not report allocation concealment reported significantly larger (10 %) reductions in infarct volume. This does not necessarily indicate that other measures to reduce the risk of bias do not have an effect, and it may be that the increase in the range of conditions tested and the observed increase in quality may be masking the identification of important determinants of outcome. Indeed, only two further variables had a significant impact on efficacy using meta-regression: we saw substantially larger effects and a robust dose-response relationship where IL-1 RA was administered centrally. A sensitivity analysis using partitioning of heterogeneity did not add substantially to our understanding.

Importantly, while the number of research teams contributing data remains somewhat limited, one included study did report data from a multilaboratory study involving eight experiments over five European centres [27].

Systematic review and meta-analysis of data from animal studies are increasingly performed and can serve a number of purposes. For instance, reviews of animal studies modelling stroke testing the efficacy of hypothermia [2] and antidepressants [28] have helped to inform the design of clinical trials including EuroHyp [4] and FOCUS [29] which are now recruiting. They can also draw attention to gaps in the quality and range of literature describing the efficacy of a particular drug that might highlight the need for further preclinical research prior to clinical trials. Of 30 instruments for assessing risk of bias of animal research, the most commonly modelled disease was stroke (9 instruments) [30], highlighting a desire to improve the translational potential of preclinical stroke research. Of ongoing interest in systematic review is the impact of individual assessment items on estimates of efficacy in large datasets, which will provide greater validity and reliability in risk of bias assessments of stroke data. Here, we have demonstrated the impact of our initial review of IL-1 RA on subsequent preclinical research and show that the efficacy originally observed has been maintained. Systematic reviews may also have an important 3Rs impact; our earlier systematic review and meta-analysis has allowed more targeted use of animals in this field and we now have a more complete picture of the usefulness of IL-1 RA for treating ischaemic stroke. The current review supports the continued investigation of IL-1 RA and identifies where efficacy remains to be verified in animals.

The limitations of this review include that our data were insufficient to perform multivariate regression using all variables of interest. This would have provided valuable information on the correlation of variables. Our analyses were prespecified, and it is possible that variables other than those investigated contributed to heterogeneity in the datasets. Additionally, as with all systematic reviews, we could only assess the impact of variables as they were reported. Not reporting blinded assessment of outcome, for example, does not necessarily mean researchers were not blinded.

To our knowledge, this is the first update to a preclinical systematic review where the changes over time in a field can be charted and the possible impacts of systematic review on the directions taken by researchers investigated. We understand from leading IL-1 RA investigators in this field that our first systematic review had a substantial, and useful, effect on their research directions. While our 2009 review was considered overly critical in many respects, the objective appraisal of range of evidence for IL-1 RA in cerebral ischaemia led researchers to address many of the evidence gaps and contributed to substantial improvements in the reporting of measures to reduce the risk of bias. In spite of the evidence for publication bias in the primary outcome measure, substantial efficacy remains, and this has been confirmed in a multicentre animal study. The major standout remaining evidence required is efficacy in hypertensive animals and in female animals. On the basis of evidence currently available, IL-1 RA is an attractive candidate drug for clinical trial.

## Electronic Supplementary Material

Below is the link to the electronic supplementary material.

Supp Figure 1 Flow diagram of search and selection of studies for inclusion depicting the number of publications included from our original review and those identified in our updated search. (DOCX 48 kb)

Supp Figure 2 Sensitivity analysis: heterogeneity in the effect of IL-1 RA on infarct volume detected using stratified meta-analysis is in part explained by (A) whether a sample size calculation was reported, (B) the route of delivery (*ICV* intracerebroventricular, *IVenous* intravenous, *SubCut* subcutaneous, *IPeritoneal* intraperitoneal), (C) dose response of centrally administered IL-1 RA, (D) anaesthetic used during induction of ischaemia, (E) type of ischaemia, (F) method of ischaemic induction, (G) whether infarct volume was corrected for oedema, (H) dose response of peripherally administered IL-1 RA and (I) time of administration. Shaded grey bars represent 95 % CI of global estimate of efficacy. Vertical error bars represent 95 % CI for individual estimates. The width of each vertical bar or size of circle reflects square root of number of animals contributing to that comparison. (GIF 528 kb)

High Resolution Image (TIF 1404 kb)

Supp Table 1 Study quality and risk of bias checklist. (DOCX 17 kb)
