# Supplementary material for: Systematic Review and Meta-Analysis of the Efficacy of Interleukin-1 Receptor Antagonist in Animal Models of Stroke: an Update
Source: Transl Stroke Res. 2016 Aug 15;7(5):395–406. doi: 10.1007/s12975-016-0489-z (PMC5014900; doi:10.1007/s12975-016-0489-z)
Supplement: Supplementary file 1 — Flow diagram of search and selection of studies for inclusion depicting the number of publications included from our original review and those identified in our updated search. (DOCX 48 kb) [file 12975_2016_489_MOESM1_ESM.docx]

Studies excluded due to full publication identified in updated search
(n = 2)

Studies excluded, with reasons:
Full text publication identified in search update (**n = 2**)

Medians reported (**n = 1**)

Total (**n = 3**)

Studies in original review
(**n = 17**)

Studies included from original review
(**n = 14**)

Studies included in analysis

Total (**n = 25**)

Full-text articles excluded, with reasons:
Conference abstract published in full (confirmed via personal communication; n = 3)

No ischaemic stroke (n = 2)

Clinical research abstract (n = 1)

No IL-1 RA treatment (n = 5)

Included in original review (n = 1)

Letter to editor (n =1)

Retracted article (n = 1)

Review article (n = 1)

No relevant outcomes (n = 1)

IL-1 RA knockout model only (n = 2)

Total (**n = 18**)

Records excluded
(**n = 405**)

Studies included from updated search
(**n = 11**)

Full-text articles assessed for eligibility
(**n = 29**)

Records screened
(**n = 434**)

## Identification

## Eligibility

## Included

## Screening

Records identified through database searching (12/02/16):

PubMed (n = 44)

Web of Science Core Collection (n =303)

BIOSIS (n = 192)

Embase (n = 56)

Total (**n = 595**)

Records after duplicates removed
(**n = 434**)

Additional records identified through other sources
Personal communication (Stuart Allan; **n = 1**)
