# Supplementary figures and images for: Systematic Review and Meta-Analysis of the Efficacy of Interleukin-1 Receptor Antagonist in Animal Models of Stroke: an Update
Source: Transl Stroke Res. 2016 Aug 15;7(5):395–406. doi: 10.1007/s12975-016-0489-z (PMC5014900; doi:10.1007/s12975-016-0489-z)

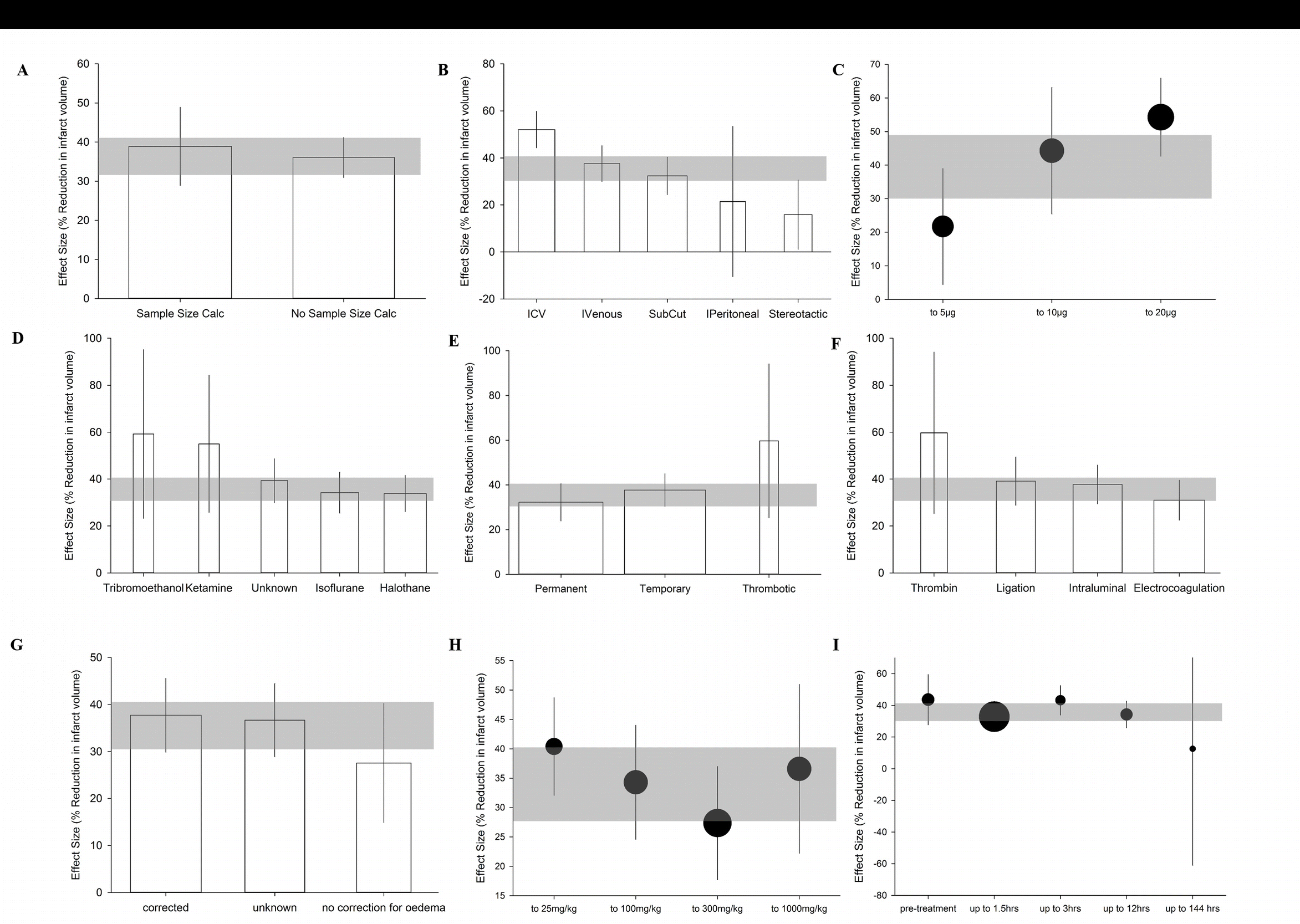

Supplement: Supplementary file 2 — Sensitivity analysis: heterogeneity in the effect of IL-1 RA on infarct volume detected using stratified meta-analysis is in part explained by (A) whether a sample size calculation was reported, (B) the route of delivery (ICV intracerebroventricular, IVenous intravenous, SubCut subcutaneous, IPeritoneal intraperitoneal), (C) dose response of centrally administered IL-1 RA, (D) anaesthetic used during induction of ischaemia, (E) type of ischaemia, (F) method of ischaemic induction, (G) whether infarct volume was corrected for oedema, (H) dose response of peripherally administered IL-1 RA and (I) time of administration. Shaded grey bars represent 95 % CI of global estimate of efficacy. Vertical error bars represent 95 % CI for individual estimates. The width of each vertical bar or size of circle reflects square root of number of animals contributing to that comparison. (GIF 528 kb) [file 12975_2016_489_Fig7_ESM.gif]

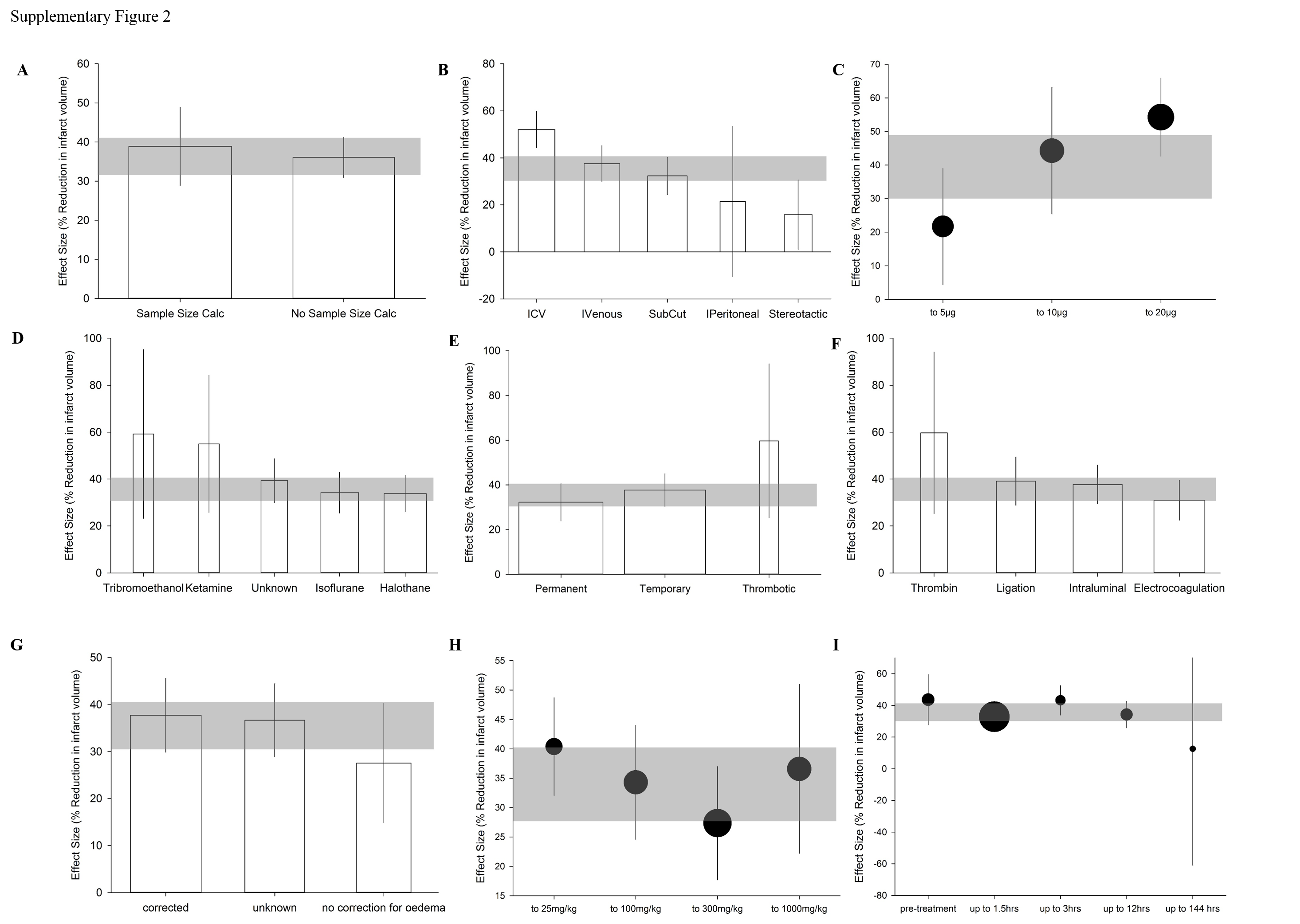

Supplement: Supplementary file 3 — High Resolution Image (TIF 1404 kb) [file 12975_2016_489_MOESM2_ESM.tif]
