# Supplementary material for: Systematic Review and Meta-Analysis of the Efficacy of Interleukin-1 Receptor Antagonist in Animal Models of Stroke: an Update
Source: Transl Stroke Res. 2016 Aug 15;7(5):395–406. doi: 10.1007/s12975-016-0489-z (PMC5014900; doi:10.1007/s12975-016-0489-z)
Supplement: Supplementary file 4 — Study quality and risk of bias checklist. (DOCX 17 kb) [file 12975_2016_489_MOESM3_ESM.docx]

| Year | Author | 1 | 2 | 3 | 4 | 5 | 6 | 7 | 8 | 9 | 10 | 11 | 12 | 13 | 14 | 15 | Total |
| --- | --- | --- | --- | --- | --- | --- | --- | --- | --- | --- | --- | --- | --- | --- | --- | --- | --- |
| 1992 | Relton | + | + |  |  |  | + |  |  |  |  |  |  |  | + |  | 4 |
| 1995 | Betz | + | + |  |  |  | + |  |  |  |  | + |  |  | + | + | 6 |
| 1995 | Garcia | + | + |  |  | + |  |  |  | + |  |  |  | + |  |  | 5 |
| 1996 | Loddick | + | + |  |  | + | + |  |  |  |  | + |  |  | + |  | 6 |
| 1996 | Relton | + |  | + | + |  | + |  |  |  |  | + |  |  |  |  | 5 |
| 1997 | Stroemer | + | + |  |  |  | + |  |  |  |  |  |  |  |  |  | 3 |
| 1997 | Yang | + | + |  |  |  | + |  |  | + |  | + |  |  | + | + | 7 |
| 1999 | Yang | + | + |  |  |  | + |  |  | + |  | + |  |  | + | + | 7 |
| 2000 | Mao | + | + |  |  |  | + |  |  |  |  | + |  |  | + | + | 6 |
| 2001 | Boutin | + | + |  |  | + | + |  |  | + |  |  |  |  |  | + | 6 |
| 2002 | Touzani | + | + |  |  |  | + |  |  | + |  |  | + |  | + | + | 7 |
| 2003 | Le Feuvre | + | + |  |  |  | + |  |  | + |  |  |  |  | + | + | 6 |
| 2003 | Mulcahy | + | + |  |  | + | + |  |  | + |  |  |  |  | + | + | 7 |
| 2003 | Tsai | + | + |  |  |  | + |  |  |  |  |  |  |  | + |  | 4 |
| 2006 | Craft | + | + |  |  |  | + |  |  | + |  |  |  |  | + |  | 5 |
| 2007 | McColl | + | + |  | + | + | + | + |  | + |  |  |  |  | + |  | 8 |
| 2008 | Clark | + | + |  |  |  | + |  |  | + |  |  | + | + | + |  | 7 |
|  | SUM | 17/17 | 16/17 | 1/17 | 2/17 | 5/17 | 16/17 | 1/17 | 0/17 | 10/17 | 0/17 | 6/17 | 2/17 | 2/17 | 13/17 | 8/17 | 6 |
|  | % | 100 | 94 | 6 | 12 | 29 | 94 | 6 | 0 | 59 | 0 | 35 | 12 | 12 | 76 | 47 | (MEDIAN) |
| 2010 | Greenhalgh | + | + | + | + | + | + |  |  | + | + |  | + | + | + | + | 12 |
| 2012 | Pradillo | + | + | + |  | + | + | + | + | + | + |  | + | + | + |  | 12 |
| 2014 | Denes | + | + | + |  | + | + | + | + | + | + |  | + | + | + | + | 13 |
| 2014 | Girard | + | + | + | + | + | + |  |  | + | + |  |  |  | + |  | 9 |
| 2014 | Xia | + |  |  |  |  |  |  |  |  | + |  |  |  |  |  | 2 |
| 2015 | Maysami | + |  | + | + | + | + | + |  | + | + |  | + | + | + | + | 12 |
| 2016 | Clausen | + | + | + | + | + | + |  |  | + |  | + |  |  | + | + | 10 |
| 2016 | Pradillo |  | + | + |  | + | + | + | + | + | + |  | + | + | + |  | 11 |
|  | SUM | 7/8 | 6/8 | 7/8 | 4/8 | 7/8 | 7/8 | 4/8 | 3/8 | 7/8 | 7/8 | 1/8 | 5/8 | 5/8 | 7/8 | 4/8 | 11.5 |
|  | % | 88 | 75 | 88 | 50 | 88 | 88 | 50 | 38 | 88 | 88 | 13 | 63 | 63 | 88 | 50 | (MEDIAN) |

**Supplementary Table 1. Study quality and risk of bias checklist**

1) Peer reviewed publication 2) control of temperature 3) randomisation to treatment or control 4) blinded induction of ischaemia 5) blinded assessment of outcome 6) avoidance of anaesthetics with marked intrinsic neuroprotective properties 7) use of animals with co-morbidities 8) sample size calculation 9) statement of compliance with animal welfare requirements 10) statement of potential conflicts of interest 11) physiological monitoring during stroke induction (in addition to control of temperature) 12) pre-specified inclusion and exclusion criteria 13) reporting of animals excluded from analysis 14) reporting of study funding and 15) injury confirmed via laser Doppler or perfusion imaging.
